# Waist circumference and pulmonary function: a systematic review and meta-analysis

**DOI:** 10.1186/2046-4053-1-55

**Published:** 2012-11-16

**Authors:** Fernando César Wehrmeister, Ana Maria Baptista Menezes, Ludmila Correa Muniz, Jeovany Martínez-Mesa, Marlos Rodrigues Domingues, Bernardo Lessa Horta

**Affiliations:** 1 Postgraduate Program in Epidemiology, Federal University of Pelotas, Rua Marechal Deodoro 1160, 3º piso, Pelotas, RS, CEP 96020-220, Brazil

**Keywords:** Anthropometry, Forced vital capacity, Forced expiratory volume, Pulmonary function tests, Review, Waist circumference

## Abstract

**Background:**

Studies have reported an impact of central obesity on people’s health. The literature is scarce on the effects of waist circumference (WC) on pulmonary function. Our objective was to review the literature on the association between WC and pulmonary function.

**Methods:**

A systematic review was carried out in the PubMed, CINAHL, Web of Science and Scopus databases. The search included published, in press and online documents up to December 2011. A meta-analysis was carried out to obtain the pooled effect, and a meta-regression was performed to evaluate sources of heterogeneity.

**Results:**

From the 547 studies identified, 10 were included. The meta-analysis revealed an inverse relationship between WC and pulmonary function parameters, indicating that the effect was greater among men (forced expiratory volume in 1 second (FEV_1_ β = −15.9 (95% confidence interval = −23.2, −8.5); forced vital capacity (FVC) β = −16.6 (95% confidence interval = −21.0, −12.2)) compared with women (FEV_1_ β = −5.6 (95% confidence interval = −9.1, −2.1); FVC β = −7.0 (95% confidence interval = −9.1, −4.8)). The meta-regression identified sex as the characteristic that most contributed to the heterogeneity (R^2^ = 54.8% for FEV_1_ and R^2^ = 85.7% for FVC).

**Conclusions:**

There seems to be an inverse relationship between WC and pulmonary function, mainly in men. More population-based studies should be performed, especially among children and adolescents, to confirm these findings.

## Background

Our lifestyle has changed in the past decades. Nowadays many of us have adopted unhealthy habits that may impair our health [1]. The global obesity rate is a result of such behaviors and is one of the main causes of chronic diseases worldwide [2]. Obesity has been associated with many chronic diseases such as cardiovascular disorders among many others; recently, some respiratory diseases and consequent loss in pulmonary function have been associated with obesity, changing dramatically overall health, life quality and lifespan [3]. Asthma is an example of a respiratory disease associated with obesity, as reported by many studies. A review by Noal and colleagues showed an association between overweight/obesity and asthma in adolescents [4].

The wide use of body mass index (BMI) as an obesity measure is explained by its simplicity, but it does not provide information on body fat distribution [5,6]. Studies have recently focused on abdominal fat accumulation and its consequences on population health. Few population-based studies using high-end equipment are published; hence, the standardized waist circumference (WC) measured by trained personnel [3,5,7-11] has been used as an estimate of abdominal fat.

The effects of obesity on pulmonary function parameters are influenced by the amount and distribution of body fat [12-14]. Studies have shown that central obesity, which may be measured by WC or by waist-to-hip ratio, can influence respiratory mechanics regardless of BMI [12,14]. Both BMI and WC are usual measures of overweight and obesity, but also indicators of body size, and they therefore may be associated with pulmonary function parameters such as forced expiratory volume in 1 second (FEV_1_) and forced vital capacity (FVC) [5,9]. The abdominal fat, measured by WC, correlates to intra-abdominal and subcutaneous adipose tissue, and is a better indicator of intra-abdominal fat (considered harmful to health) than BMI [15]. Obesity-related health risks are better explained by WC than BMI [16], as the WC provides information on fat distribution that cannot be obtained from BMI [15]. Besides, WC may affect ventilatory mechanics because it limits diaphragm expansion [6].

A review study on physiology of obesity and its effects on pulmonary function showed that central obesity is more likely to affect pulmonary volumes, without direct effects on pulmonary obstruction [6]. The aim of the present review is therefore to evaluate the association between WC and pulmonary function parameters reporting a combined effect measure through a meta-analysis.

## Methods

### Search strategy

A systematic review was carried out using independent keywords in the following electronic databases: PubMed, Web of Science, Scopus and CINAHL. The use of independent keywords allows a broader search as it rules out potential mistyping and other errors when using Mesh Terms (Medical Subject Headings) in different databases. The keywords used were: (‘waist circumference’ OR ‘abdominal circumference’ OR ‘central obesity’ OR ‘abdominal obesity’ OR ‘waist-hip ratio’) AND (‘spirometry’ OR ‘lung function tests’ OR ‘pulmonary function tests’ OR ‘respiratory function tests’ OR ‘forced expiratory volume’ OR FEV OR ‘forced vital capacity’ OR FVC OR ‘peak expiratory flow rate’ OR PEFR). In the Web of Science database, we needed to include ‘TS=’ before the parentheses for each subset of terms. The search was carried out on 1 January 2012 including all papers published until the end of 2011, with no data limits.

### Criteria for inclusion and exclusion of papers

To be included in the review, papers should be based on population-based observational (cross-sectional or cohort) studies and should report the association between WC and pulmonary function parameters. Studies among specific groups (twins or people affected by a specific illness) were not included. Studies reporting only the peak expiratory flow rate assessed by peak flow and not by spirometer and that did not use linear regression during analysis were excluded from the present review.

### Stages of reference selection

All references were imported into Endnote software (EndNote X3; Thompson Reuters Inc. Philadelphia, PA, USA). One of the authors (FCW) later read the titles and excluded those that did not report the outcomes of interest. Two authors (FCW and LCM) then read all abstracts independently. All disagreements with respect to inclusion/exclusion of papers were judged by a third author (JM-M). Independent reading of full texts was again done by two reviewers (FCW and LCM), while a third reviewer was used when disagreements happened. Information from papers was retrieved by two independent referees (FCW and LCM) and occasional disagreements were decided by the third reviewer (JM-M). After this stage, the list with all references from the selected papers was examined to look for other references that have not yet been included.

### Data extraction

Characteristics of studies, such as sample size, design, country, WC and pulmonary function measurement procedures, were extracted from papers. We also extracted linear regression coefficients (β) and dispersion measures (standard error and standard deviation, when available, or upper/lower bounds of 95% confidence intervals (CIs)). The data extraction was carried out by two independent reviewers (FCW and LCM) and divergences were solved by consensus.

### Evaluation of selected papers

All papers were classified according to an adaptation of the Downs and Black checklist [17]. From the 27 original items in the checklist, 17 were employed as follows. (Item 1) Is the hypothesis/aim/objective of the study clearly described? (Item 2) Are the main outcomes to be measured clearly described in the Introduction or Methods section? (Item 3) Are the characteristics of the patients included in the study described clearly? (Item 4) Are the distributions of principal confounders in each group of subjects to be compared described clearly? (Item 5) Are the main findings of the study described clearly? (Item 6) Does the study provide estimates of the random variability in the data for the main outcomes? (Item 7) Have the characteristics of patients lost to follow-up been described? (Item 8) Have actual probability values been reported (for example, 0.035 rather than <0.05) for the main outcomes except where the probability value is less than 0.001? (Item 9) Were the subjects asked to participate in the study representative of the entire population from which they were recruited? (Item 10) If any of the results of the study were based on ‘data dredging’, was this made clear? (Item 11) Were the statistical tests used to assess the main outcomes appropriate? (Item 12) Were the main outcome measures used accurate (valid and reliable)? (Item 13) Were the patients in different groups recruited from the same population? (Item 14) Were study subjects recruited over the same period of time? (Item 15) Was there adequate adjustment for confounding in the analyses from which the main findings were drawn? (Item 16) Were losses of patients to follow-up taken into account? (Item 17) Did the study have sufficient power to detect a clinically important effect where the probability value for a difference being due to chance is less than 5%?

Each item scored one point, except for Item 4 that could result in 0 (no), 1 (partially) and 2 (yes). The scoring could therefore range from 0 to 18 points. Papers were categorized as: high chance of bias (0 to 5 points), average chance of bias (6 to 11 points) and low chance of bias (12 to 18 points). Papers were scored independently by two referees and occasional disagreements were decided by the third reviewer.

### Meta-analysis

A meta-analysis was carried out with data on FEV_1_ and FVC. To obtain the pooled effect, a random model [18] was employed in all analysis, due to the high heterogeneity observed (*I*^2^ >90%) and possible differences in the measurement of WC. Only studies that measured the association between WC as a continuous variable with absolute values for FEV_1_ and FVC were included. As linear regression was used in all studies, those presenting results in liters were changed into milliliters. When the results of the study were not in accordance with such demands (WC in percentiles or predicted values for FEV_1_ and FVC), the authors were contacted by email (three attempts) to obtain information to enable inclusion of the study in the meta-analysis. Two out of four authors replied.

For meta-analysis purposes, we pooled the regression coefficients (β) of the association investigated. During meta-analysis, studies presenting results stratified by sex were included twice, as independent analysis. After contact with the correspondence author of one study [3], the regression coefficients of the association between WC and FEV_1_ and FVC, which were in quintiles, were changed into centimeters, dividing each quintile by its respective increments (6.3 cm in men and 6.9 cm in women), because according to the author the relationship was linear. The only study among children and adolescents [9] was not included in the meta-analysis. Lastly, a meta-regression was performed to assess the contribution of some variables to the heterogeneity between studies. In the meta-regression, besides variables from the study (sex, WC measurement method, sample size and age, and statistical adjustment for height, weight and age), other variables that could potentially explain the results’ heterogeneity were included: geographical continent where the study was carried out and the Human Development Index, obtained previously and/or during the year of the research.

## Results

**Figure 1:**
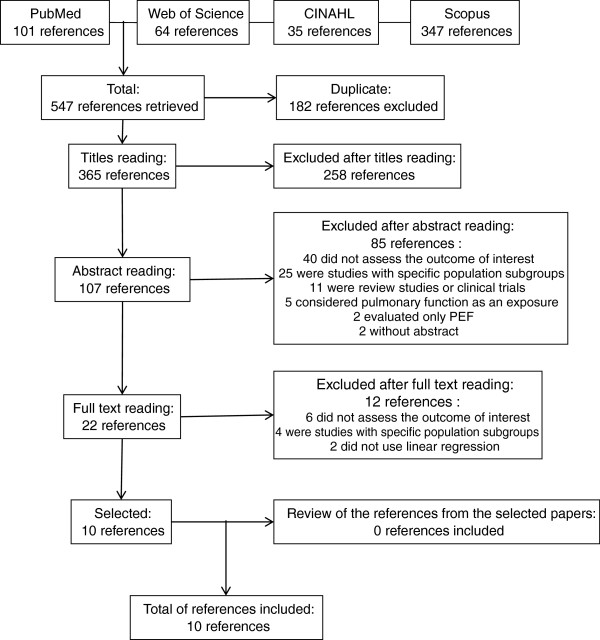
Flow chart of paper selection for the review.

A total of 547 references were retrieved: 101 from PubMed, 64 from Web of Science, 35 from CINAHL and 347 from Scopus. From these 547 references, 182 were duplicated – leaving 365 titles to be read. After the selection stages, 10 papers were included in the review. All stages of reference selection, and numbers of papers included/excluded are displayed on the flow chart (Figure 1). Additional information can be visualized in Additional file 1.

The details of each study are shown in Table 1. The 10 studies selected for analysis were published between 1999 and 2011, and seven were cross-sectional (Table 1). Sample size in the studies ranged from 718 [9] to 21,550 [3] individuals. Most studies included individuals older than 18, and only one study involved children and adolescents [9]. Only one study from a developing country was found [19]. All pulmonary function parameters were measured by spirometry, while waist circumference was measured with tape by trained staff. However, distinct body sites were used to locate and measure waist circumference. Depending on the study, measurements were taken on the navel line, the smallest circumference or the midpoint between the ribs and iliac crest. Seven out of 10 studies included presented sex-stratified analysis. Based on the Downs and Black checklist, all papers scored >12, indicating a low chance of bias.

**Table 1:** **Overview of studies included in the systematic review (*n *= 10)**

**Author**, **year, place**	**Age (years)**	**Design**	**Waist circumference measurement method**	**Sample**	**Sex**	**Effect**	**Adjustments**
Canoy and colleagues, 2004 [3], UK	45 to 79	Cohort with cross-sectional analysis	Smallest circumference between the ribs and iliac crest (quintiles)	9,674	Male	FEV_1_ (ml): β = −120.3 (95% CI = −134.6, –106.1). FVC (ml): β = −129.1 (95% CI = −147.9. –110.2)	Age, height, BMI
				11,876	Female	FEV_1_ (ml): β = −53.2 (95% CI = −62.0, –44.5). FVC (ml): β = −60.5 (95% CI = −71.7, –49.2)	
Carey and colleagues, 1999 [7], UK	18 to 73	Cohort	Midpoint between the ribs and iliac crest (difference in standard deviations of the measure in different follow-ups)		Male	ΔFEV_1_ (ml): 18 to 45 years, β = −30.0 (SE 12.0); 46 to 73 years, β = −53.0 (SE 14.0)	Delta FEV_1_ adjusted for height and age
					Female	ΔFEV_1_ (ml): 18 to 45 years, β = −42.0 (SE 10.0); 46 to 73 years, β = −30.0 (SE 11.0)	
Chen and colleagues, 2001 [8], UK	25 to 64	Cross-sectional	Midpoint between the ribs and iliac crest (centimeters)	865	Male	FEV_1_ (l): β = −0.017 (SE 0.004). FVC (l): β = −0.008 (SE 0.004)	Height, age, weight, current occupation, caloric intake and smoking
				971	Female	FEV_1_ (l): β = −0.009 (SE 0.002). FVC (l): β = −0.007 (SE 0.003)	
Chen and colleagues, 2009 [9], Canada	6 to 17	Cross-sectional	Smallest circumference between the ribs and iliac crest (centimeters)	718	Both	FEV_1_ (l): β = 0.002 (SE 0.001). FVC (l): β = 0.004 (SE 0.002). Relation FEV_1_/FVC: β = −0.053 (SE 0.027)	Sex, age, weight and height
Chen and colleagues, 2007 [5], Canada	18 to 79	Cross-sectional	Smallest circumference between the ribs and iliac crest (centimeters)	1,674	Both	FEV_1_ (l): β = −0.011 (SE 0.002). FVC (l): β = −0.013 (SE 0.002). Relation FEV_1_/FVC: β = −0.031 (SE 0.025)	Sex, age, height, weight and smoking
Choi and colleagues, 2011 [21], South Korea	≥19	Cross-sectional	Midpoint between the ribs and iliac crest (centimeters)	1,059	Male	FVC (% predicted): β = −0.17 (SE 0.059). Relation FEV_1_/FVC: β = 0.0007 (SE 0.0005)	Smoking, blood glucose, systolic blood pressure, total cholesterol, triglyceride and HDL
				1,555	Female	FVC (% predicted): β = −0.15 (SE 0.04). Relation FEV_1_/FVC: β = −0.0001 (SE 0.002)	
Ochs-Balcom and colleagues, 2006 [11], USA	35 to 65	Cross-sectional	Smallest circumference between the ribs and iliac crest (centimeters)	985	Male	FEV_1_ (% predicted): β = −0.233 (SE 0.04). FVC (% predicted): β = −0.350 (SE 0.04). Relation FEV_1_/FVC: β = 0.089 (SE 0.02)	Smoking, schooling and eosinophils. FEV_1_/FVC ratio adjusted for age, height and skin color too
				1,168	Female	FEV_1_ (% predicted): β = −0.086 (SE 0.03). FVC (% predicted): β = −0.176 (SE 0.03). Relation FEV_1_/FVC: β = 0.086 (SE 0.02)	
Paek and colleagues, 2010 [22], South Korea	≥19	Cross-sectional	Smallest circumference between the ribs and iliac crest (centimeters)	4,001	Both	FEV_1_ (% predicted): β = −0.21. FVC (% predicted): β = −0.13. Relation FEV_1_/FVC: β = −0.029	Relation WC/height, age, sex, smoking, physical activity, alcohol consumption and socioeconomic status
Steele and colleagues, 2009 [20], UK	40.6 (mean)	Cohort with cross-sectional analysis	Midpoint between the ribs and iliac crest (centimeters)	238	Male	FEV_1_ (l): β = −0.02 (95% CI = −0.02, –0.01). FVC (l): β = −0.025 (95% CI = −0.032, –0.018)	Age, height and smoking
				364	Female	FEV_1_ (l): β = −0.004 (95% CI = −0.008, –0.001). FVC (l): β = −0.004 (95% CI = −0.009, 0.001)	
Ubilla and colleagues, 2008 [19], Chile	21 to 28	Cohort with cross-sectional analysis	Navel line (terciles, middle as reference)	550	Male	FEV_1_ (ml): lower tercile, β = 93.1 (95% CI = 14.4, 200.6); upper tercile, β = −76.9 (95% CI = −183.2, 29.5). FVC (ml): lower tercile, β = 80.7 (95% CI = 43.0, 204.4); upper tercile, β = −98.8 (95% CI = −221.2, 23.6)	Age, height, smoking, number of siblings, schooling and gestational age. Nutritional variables throughout life, except BMI
				671	Female	FEV_1_ (ml): lower tercile, β = −62.1 (95% CI = −132.1, 7.9); upper tercile, β = −22.1 (95% CI = −93.7, 49.5). FVC (ml): lower tercile, β = −96.2 (95% CI = −177.1, –15.2); upper tercile, β = −42.5 (95% CI = −125.3, 40.3)	

BMI, body mass index; CI, confidence interval; FEV_1_, forced expiratory volume in 1 second; FVC, forced vital capacity; HDL, high-density lipoprotein; SE, standard error; WC, waist circumference.

### Association of waist circumference with FEV_1_

FEV_1_ was analyzed as a continuous variable and as percentage of the predicted value. All studies, except the one with children and adolescents [9], presented an inverse relationship between WC and FEV_1_; however, this relationship was not statistically significant in all studies (Table 1). Canoy and colleagues and Ubilla and colleagues used quintiles and terciles, respectively, to evaluate the association of WC with FEV_1_[3,19]. In the study by Canoy and colleagues, carried out in the UK among adults, each step from one WC quintile to another represented a decrease in FEV_1_ of 120.3 ml (95% CI = −134.6, –106.1) in men and 53.2 ml (95% CI = −62.0, −44.5) in women [3] (Table 1). On the other hand, the study by Ubilla and colleagues showed no association of WC (terciles) with FEV_1_ for men or women [19]. When treated as a continuous variable, the effect of a 1-cm increase in the WC resulted in a decrease of 4 ml in women [20] to 20 ml in men [20] (Table 1). Only one cohort study presented longitudinal analysis [7] – showing that the larger the WC difference (in standard deviations) from one follow-up to the other, the lower the FEV_1_.

### Association of waist circumference with FVC

The review showed an inverse relationship also between WC and FVC, except for children and adolescents [9]. The association of WC with FVC, however, seems to be larger than that for FEV_1_. When the FVC was studied as continuous information, a 1-cm increment in WC decreased FVC by 13 ml in both sexes [5] (Table 1). However, studies presenting sex-stratified analysis showed that the decrease in FVC (absolute values) was higher in men than women for the WC increase as a continuous variable [8,20] or in quintiles [3] (Table 1). The FCV analyzed as a percentage of predicted values also presented an inverse relationship with WC; a similar pattern was observed with absolute values. In men, as the WC increased the predicted FVC value decreased from 0.170 [20] to 0.350 [11]. In women, this decrease ranged from 0.150 [21] to 0.176 [11] (Table 1).

### Association of waist circumference with the FEV_1_/FVC relation

From the 10 studies in this review, five presented results for the association between WC and the FEV_1_/FVC relation, but only two showed statistically significant results [11,22], both in adults. Paek and colleagues observed, in South Korea, a reduction in the FEV_1_/FVC relation of 0.029% for each 1-cm increase in WC, adjusted for sex, age, weight and other covariates [22] (Table 1). On the contrary, Ochs-Balcom and colleagues presented sex-stratified results and, different to FEV_1_ and FVC, the effect was the same among men and women [11] (Table 1).

### Meta-analysis and meta-regression

**Figure 2:**
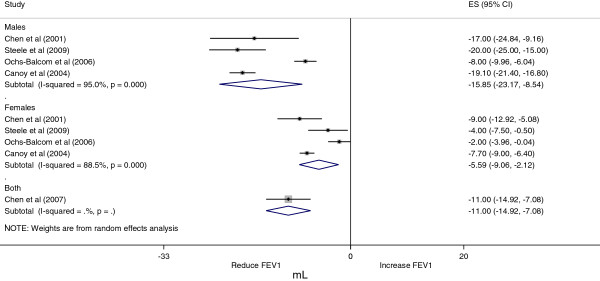
**Grouped effect for association between waist circumference and forced expiratory volume among adults.** Sex-stratified effect of the association between waist circumference (WC) and forced expiratory volume in 1 second (FEV_1_) among adults.

**Figure 3:**
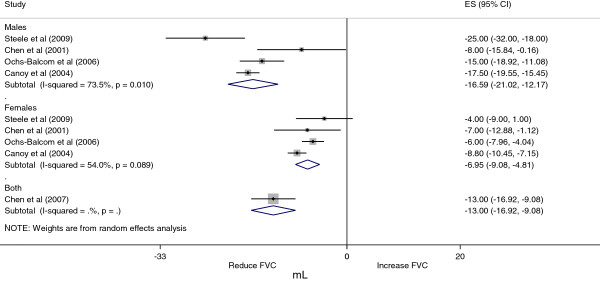
**Grouped effect for association between waist circumference and forced vital capacity among adults.** Sex-stratified effect of the association between waist circumference (WC) and forced vital capacity (FVC) among adults.

The meta-analysis was carried out with FEV_1_ and FVC values. The pooled effect of the association between WC and FEV_1_ is displayed in Figure 2 and the association between WC and FVC is shown in Figure 3. We decided to present only sex-stratified effects due to an apparent difference in the association according to sex. Despite the small number of studies in the meta-analysis, the effect in men (FEV_1_ β = −15.9 (95% CI = −23.2, –8.5); FVC β = −16.6 (95% CI = −21.0, –12.2)) presented larger magnitude compared with that in women (FEV_1_ β = −5.6 (95% CI = −9.1, –2.1); FVC β = −7.0 (95% CI = −9.1, –4.8)). The FEV_1_/FVC relation was not used in the meta-analysis due to the very low number of studies showing these results (only three out of five studies met the inclusion criteria).

When the individual contribution of each characteristic in the heterogeneity by meta-regression (Table 2) was analyzed, we observed that including sex in the model resulted in a reduction of 54.8% for FEV_1_ and 85.7% for FVC. Other tested variables were not important to lower the heterogeneity of the results during meta-analysis (Table 2).

**Table 2:** Individual contribution of each variable in the decrease of heterogeneity, measured by meta-regression

	**Adjusted *R***^**2**^**(%)**	
**Variable**	**FEV**_**1**_	**FVC**
Sex^a^	54.8	85.7
Waist circumference measurement method	0.0	0.0
Age	0.0	0.0
Sample	0.0	0.0
Adjustment for height, weight and age	0.0	0.0
Study quality (modified Downs and Black scale)	0.0	0.0
Human Development Index	10.2	0.0
Geographical continent where study was carried out	7.7	0.0

FEV_1_, forced expiratory volume in 1 second; FVC, forced vital capacity. ^a^For this analysis, the study reporting measurements for both genders [5] was not included.

The reasons for exclusion of papers from the meta-analysis were: pulmonary function measurement expressed as time change [7] or as percentage of predicted value [21,22], WC in terciles [19] or a study with children [9].

## Discussion

The present review highlights a potential negative relationship between WC and pulmonary function, especially with respect to FEV_1_ and FVC. The clinical relevance of these findings must be interpreted with caution due to the few number of studies included in the present meta-analysis.

The major strengths of this review are the exclusive inclusion of population-based studies, improving external validity of the results. Another interesting aspect is the use of linear regression, which allowed us to establish the amount of pulmonary function reduction for each increase in waist circumference; this would not be possible if we included studies using cutoff points for pulmonary function parameters.

One possible limitation is the use of different spirometers among the studies. Nevertheless, most of the reviewed studies used portable spirometers; the paper from Liistro and colleagues shows a good reproducibility for FEV_1_ using 10 different portable spirometers in a multicenter study [23]; regarding FVC, some of the spirometers showed large confidence intervals. We therefore cannot rule out a potential bias for some of the lung function parameters, but if there is such bias it is conservative.

It is important to observe that nearly all included studies were cross-sectional (or with cross-sectional analysis) and evaluated adults (18 years or older). In addition, only one study was carried out in a developing country [19], indicating the need for future studies in these countries.

The prevalence of obesity and body shapes may differ between developed and poorer countries. The study by Ford and colleagues points out an increase in abdominal obesity, measured by WC, in the USA [24], while other countries do not have enough data to draw such conclusion. Other factors that may influence the WC measure are age, sex, physical activity, skin color and overall adiposity [15], all characteristics that change drastically among different countries. The effect of WC on FEV_1_ and FVC in children and adolescents could not be assessed in the present review because only one Canadian study evaluated this age group [9]. In contrast to what was observed in older people and adults, a direct relationship between WC and pulmonary function parameters (FEV_1_ and FVC) was observed in this study, possibly due to the growth process that occurs during adolescence, as WC is considered an indication of body size, consequently influencing pulmonary volumes in children and adolescents.

Klein and colleagues attempted to establish a geometrical relationship between weight, BMI and WC (considered as the circumference of a cylinder) [15]. WC mirrors body shape, while BMI provides an estimate of body mass and volume [15]. Different measures of adiposity such as indices and others (Dual-energy X-ray Absorptiometry, air displacement plethysmography, bioimpedance among others) have been used in epidemiological studies. However, some of these measures are very expensive and cannot be used in most of the studies. In the present review, we observed that WC was measured differently according to the study, which could influence the magnitude of the association with pulmonary function parameters. However, the meta-regression results show that the WC measurement method could not explain the heterogeneity of results. Wang and colleagues report 14 different sites to measure WC, all between the 10th rib and iliac crest [25]. Klein and colleagues describe five main methods to measure WC in clinical settings or in epidemiologic studies: midpoint between the last rib and iliac crest; navel level; wider or narrower waist circumference; right below the rib cage; and right above the iliac crest [15].

Owing to discrepancy in measuring the WC, Wang and colleagues carried out a study in the USA to establish which WC measuring method was the most accurate to predict abdominal fat [25]. The researchers measured the WC in 111 individuals (adults) using the following sites: narrower circumference in the midpoint between the last rib and iliac crest, and immediately below the lowest rib and right above the iliac crest. The authors repeated the measurement three times in a subgroup (*n* = 98). All four methods were highly correlated (*r* >0.99) in men and women. They also provided reliable information on the trunk fat percentage, especially in women (*R*^2^ >0.65 in women and *R*^2^ <0.45 in men). Klein and colleagues thus discuss that there is no best site for measuring the WC [15]. In this review, apparently, there are no differences in the magnitude of the association between WC with FEV_1_ or FVC regardless of the measuring method to obtain the WC.

Obese people, especially those with severe obesity, have higher metabolic demands and also increased respiratory demands [13]. Much has been said about the disadvantages of BMI to measure body fat, especially because it measures body density. New indexes are therefore considered best for evaluating fat [26] and fat distribution in the body [15]. As previously known, obesity mainly when measured by BMI is not a good parameter to evaluate body fat distribution [6,26]. Abdominal obesity, measured by WC, was initially discussed as a risk factor for cardiovascular mortality and morbidity. Nowadays, other diseases are being studied to investigate the association with central obesity, including conditions related to pulmonary function [27]. One of the most discussed explanations in the literature is that abdominal fat may interfere in pulmonary mechanics, causing restrictions during breathing, potentially reducing respiratory volumes, such as FEV_1_ and FVC [6,13,28]. This mechanical effect is more evident if central obesity is considered instead of overall or peripheral fat [10,12].

The excess of fat in the abdomen and thoracic region may lead to decreases in the compliance and resistance of respiratory system, increasing energetic demands of breathing [28]. Measuring the compliance of the respiratory system is a hard task, however, as it must be relaxed and inactive to provide an accurate measure [6]. Another potential mechanism is that the increase in WC may have an effect on the diaphragm, limiting its movements [6]. There is no difference in the effect size on the respiratory system when different abdominal obesity indicators are used, such as WC and waist-to-hip ratio, and thoracic fat indicators [6], suggesting an interdependency of these measures with respect to pulmonary function parameters.

The intention to run the analysis stratified according to potential heterogeneity sources was only workable for sex as other variables were not present in most studies, resulting in very few studies to be analyzed. The meta-analysis is very difficult to perform because not all studies present the same units to measure both WC (measured in centimeters, standard deviations or percentiles) or pulmonary function parameters (measured as absolute values and percentage of the predicted values).

During the evaluation of the high heterogeneity in our findings, indicated by the high *I*^2^ value in the meta-analysis, intrinsic factors to the study and some associated with the country where the research was carried out were assessed by the meta-regression. Heterogeneity was better explained by sex: *R*^2^ = 54.8% for men and *R*^2^ = 85.7 for women. It is known that the main determinants of pulmonary function are age, height and sex [29]. Another factor that could explain the difference in the magnitude of the association between WC with pulmonary function is the body fat accumulation pattern. In women, fat accumulates around the hips; while in men the abdomen is usually where fat is accumulated [30].

As a consequence, perhaps, the effect of WC on pulmonary function in men tends to be larger than that in women. The Human Development Index explained 10.2% of heterogeneity of the results. One plausible explanation is that the Human Development Index and physical activity are positively correlated [31], and physical inactivity is a major risk factor for obesity and other health conditions, most of them also associated with WC increases.

## Conclusions

Increases in WC result in decreased pulmonary function parameters, such as FEV_1_ and FVC in people older than 18 years, but it does not affect the relation between the two parameters. We also identified that the effect of WC on pulmonary function parameters was greater among men compared with women. Central obesity is possibly associated with restrictive patterns, but not airways obstruction; however, it cannot be entirely ruled out. Longitudinal studies, especially among children and adolescents, are needed to verify the effects in these age groups, as well as the long-term effects of WC on pulmonary function.

## Abbreviations

BMI: body mass index; CI: confidence interval; CINAHL: Cumulative Index to Nursing and Allied Health Literature; FEV_1_: forced expiratory volume in 1 second; FVC: forced vital capacity; WC: waist circumference.

## Competing interests

The authors declare that they have no competing interests.

## Authors’ contributions

FCW had the idea for the study, reviewed all papers included in the review, performed the meta-analysis and drafted the manuscript. AMBM participated in the design of the study and contributed to the writing of the manuscript. LCM and JM-M reviewed the papers included in this review and helped in the data analysis. MRD and BLH helped with the meta-analysis and critically reviewed the manuscript. All authors read and approved the final manuscript.

## Supplementary Material

Additional file 1 PRISMA 2009 flow diagram. Click here for file
