# Supplementary material for: Waist circumference and pulmonary function: a systematic review and meta-analysis
Source: Syst Rev. 2012 Nov 16;1:55. doi: 10.1186/2046-4053-1-55 (PMC3534560; doi:10.1186/2046-4053-1-55)
Supplement: Additional file 1 — PRISMA 2009 flow diagram. [file 2046-4053-1-55-S1.doc]

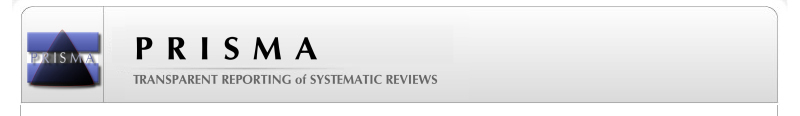
**PRISMA 2009 Flow Diagram**

**Screening**

**Included**

**Eligibility**

**Identification**

Records identified through database searching
(n = 547 )

Additional records identified through other sources
(n = 0 )

Records after duplicates removed
(n = 365 )

Records screened
(n = 107 )

Records excluded
(n = 85 )

Full-text articles assessed for eligibility
(n = 22 )

Full-text articles excluded, with reasons
(n = 12 )

6 did not assess the outcome of interest

4 were studies with specific population subgroups

2 did not use linear regression

Studies included in qualitative synthesis
(n = 10 )

Studies included in quantitative synthesis (meta-analysis)
(n = 5 )
